# One-dimensional Growth of Zinc Crystals on a Liquid Surface

**DOI:** 10.1038/srep19870

**Published:** 2016-01-29

**Authors:** Chenxi Lu, Yi Cheng, Qifa Pan, Xiangming Tao, Bo Yang, Gaoxiang Ye

**Affiliations:** 1 Department of Physics, Zhejiang University, Hangzhou 310027, P. R. China; 2 Department of Physics, Zhejiang University of Science & Technology, Hangzhou 310023, P. R. China

## Abstract

The catalyst-free growth of nanocrystals on various substrates at room temperature has been a long-standing goal in the development of material science. We report the growth of one-dimensional zinc nanocrystals on silicone oil surfaces by thermal evaporation method at room temperature (20 ± 2 °C). Uniform zinc nanorods with tunable size can be obtained. The typical length and width of the nanorods are 250–500 nm and 20–40 nm, respectively. The growth mechanism can be attributed to the effect of the liquid substrate and the preferential growth direction of the crystals. This result provides a novel and simple way to fabricate the precursors (zinc crystals) for preparation of Zn-based semiconductors and other metallic crystals on liquid substrates.

Studies on the growth of crystals on solid substrates have been made great progress in the last few decades^1^,^2^,^3^. There also have been some noticeable research achievements of the growth of organic molecular crystals in/on liquid substrates. For example, M. Voigt *et al*.^4^ reported the nucleation and growth of tetracene crystals in a thin liquid film in 2003. Subsequently, X. D. Liu *et al*.^5^ successfully prepared perylene crystals on a liquid substrate. However, there are few reports on the growth of metallic crystals, especially one-dimensional crystals on liquid surfaces, although abundant investigations on the nucleation and aggregation of metal atoms on liquid substrates have been performed^6^,^7^,^8^,^9^.

Generally, nucleation and aggregation processes of metallic atoms on liquid surfaces follow the two-stage growth model^6^. Since the liquid surfaces possess an isotropic characteristic and can be considered as quasi-free sustained substrates, the metallic atoms on the liquid substrates may diffuse randomly with large diffusion coefficients, compared with that on solid substrates^6^. Therefore the metallic atoms always trend to form compact clusters and ramified aggregates with amorphous or polycrystalline microstructures^10^,^11^.

On the other hand, the vapor-liquid-solid (VLS) mechanism^12^ and solution-liquid-solid (SLS) mechanism^13^ are two typical growth mechanisms of one-dimensional nanostructures on the liquid-solid interface, which have been widely applied to guide the growth of zinc (Zn) oxide nanowires^14^,^15^, carbon nanotubes^16^,^17^, nanowires of elemental semiconductors^18^,^19^ and compound semiconductors^20^,^21^ etc. However, appropriate catalysts and high reaction temperature are required in these growth processes.

We report a one-dimensional growth of Zn crystals on silicone oil surfaces by thermal evaporation method at room temperature. Our results show that the Zn crystals, i.e., the Zn nanorods, can grow on the oil surface along [002] direction, which is the preferential growth direction of the hexagonal structure crystal. As a result, various Zn nanorods with different length, width and thickness can be fabricated. It is remarkable that such growth process is catalyst-free and can be achieved at room temperature.

## Results

### Morphologies of the Zn crystals

**Figure 1:**
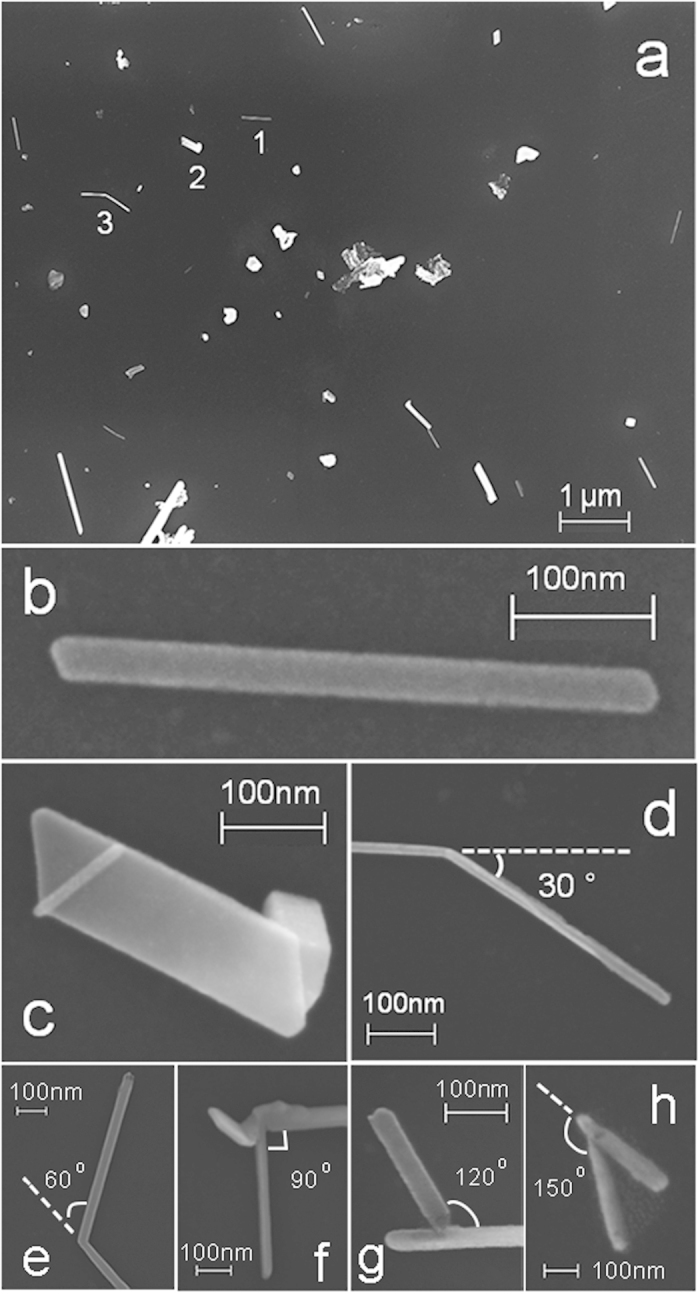
**SEM images of Zn nanorods grown on silicone oil surfaces ( *****f*** = **0.01 nm/s,** ***d*** = **8.0 nm).** (**a**) overview of the Zn nanorods in 10.0 × 7.8 μm^2 ^area and (**b**–**d**) corresponding to the Zn nanorods marked with 1, 2 and 3 in (**a**), respectively. (**e**–**h**) are corresponding to the polyline Zn crystals with drift angles of 60, 90, 120 and 150 degree, respectively.

An unexpected and interesting result is visualized in Fig. 1a, where it can be seen that the deposited Zn atoms formed massive Zn nanorods on the oil surface, indicating that a one-dimensional aggregation of the Zn atoms indeed happened on the oil surface. Obviously, this experimental result cannot be explained by the two-stage growth model^6^. It is noted that the nanorods are distributed randomly on the oil surface, due to the isotropic characteristic of the liquid surface.

From Fig. 1b,c, we can see that the Zn nanorods generally possess distinct tips with corner angles of 120 or 60 degrees, which provides an important hint of the Zn crystal growth on the oil surfaces. In our experiment, most of the Zn crystals (>95%) are uniform nanorods (i.e., straight rods with parallel edges). However, a few exceptions with particular morphologies are also observed, such as needle-like, curve shape and polyline Zn crystals etc. Figure 1d demonstrates a polyline Zn crystal which changes its growth orientation with 30 degree drifting off the original direction. Besides, nanorods (polyline Zn crystals) with drift angles of 60, 90, 120 and 150 degree, as shown in Fig. 1e–h, are also observed in our experiment. Basically, the drift angles are corresponding to the angles between different crystal planes. It is suggested that several experimental factors may contribute to this phenomenon, such as the defect movement in the crystals, local asymmetrical distribution of Zn atoms, impurities on the oil surface etc.

If take a close look at the scanning electron microscope (SEM) images shown in Fig. 1, we find that each nanorod exhibits a characteristic brightness, which indicates the specific thickness of the nanorod. For example, the brightnesses of nanorod 1 and 2 marked in Fig. 1a and their corresponding high-resolution SEM images (Fig. 1b,c) show that the thickness of nanorod 1 is smaller than that of nanorod 2.

### Statistical distribution of the length and width of the Zn nanorods

**Figure 2:**
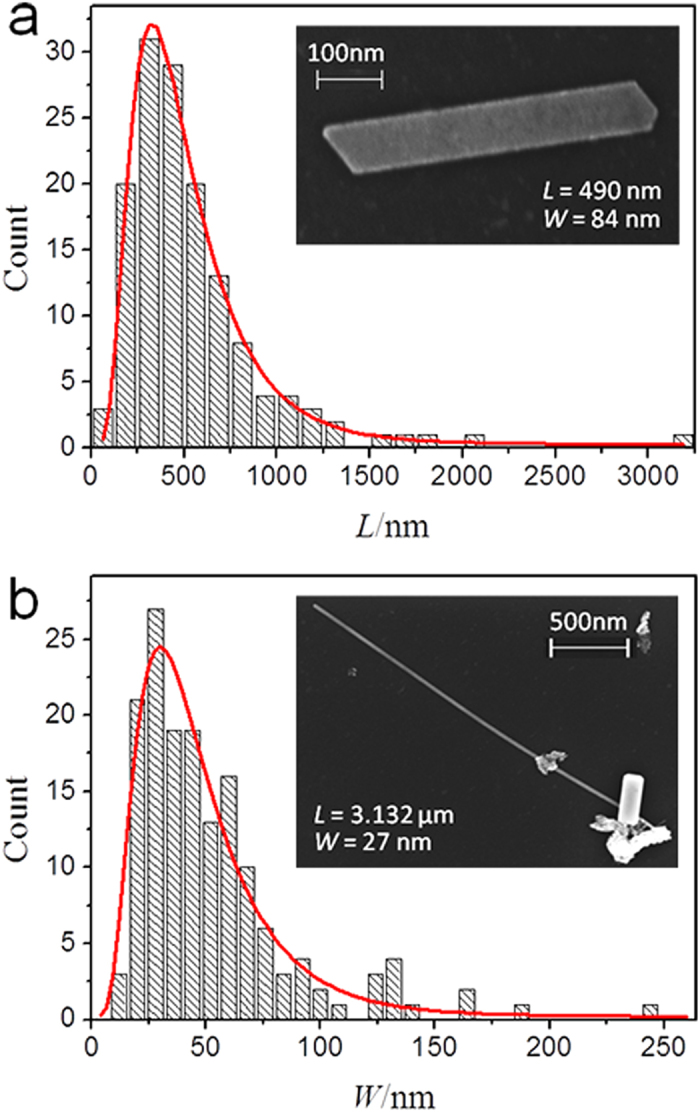
Statistical distribution of the length and width of the Zn nanorods. (**a**,**b**) show the length (*L*) and width (*W*) distributions of the Zn nanorods, respectively. And the insets in (**a**,**b**) serve as specific cases corresponding to the most probable length and width ranges, respectively. The solid curves are the lognormal distribution fittings to the experimental data.

By measuring over 140 Zn nanorods from randomly recorded SEM images, the statistical distribution of the length and width is plotted in Fig. 2a,b, with the most probable length and width ranges of 250–500 nm and 20–40 nm, respectively. Figure 2a indicates that the length of the majority of the nanorods is less than 1500 nm. A sample with a length (*L*) of 490 nm and width (*W*) of 84 nm is presented in the inset of Fig. 2a. Figure 2b shows that the width of the majority of the nanorods is less than 120 nm. As one of the longest nanorods observed in our experiment, the nanorod exhibited in the inset of Fig. 2b, with *L* = 3.132 μm, *W* = 27 nm and 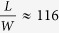, is slightly curving. Actually, it can be called a nanowire rather than a nanorod. If the width of a Zn nanorod is large enough, it approaches a two-dimensional structure as shown in Fig. 1c.

Let’s suppose that the full width at half-maximum of the length distribution is *L*_*m*_ and the full width at half-maximum of the width distribution is *W*_*m*_. From the distribution curves plotted in Fig. 2, the ratio 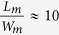, which represents the intensity of the one-dimensional growth tendency at temperature *T*. Remarkably, both *L*_*m*_ and *W*_*m*_ are relatively small, which is beneficial to fabricate uniform nanorods with a slight fluctuation in length and width.

### The compositions of the nanorods

**Figure 3:**
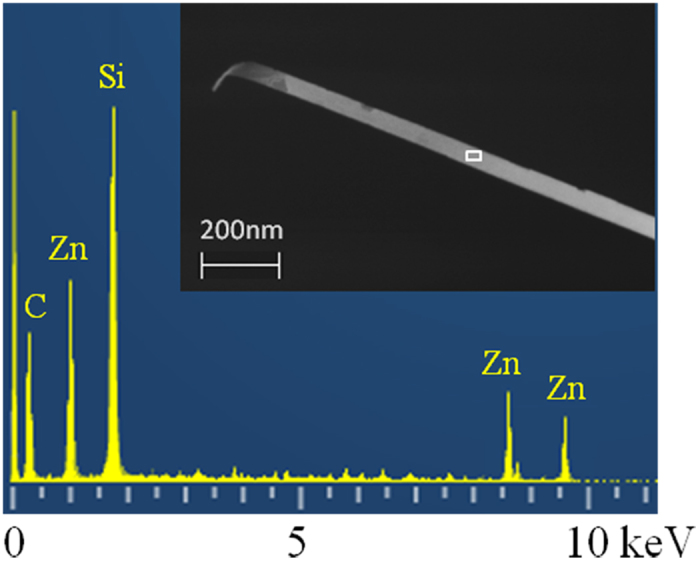
EDS spectrum of the white rectangle region of the nanorod shown in the inset. The spectrum is presented with log scale on the y-axis.

The compositions of the samples were investigated by EDS (energy dispersive X-ray spectroscopy). The EDS spectrum, as shown in Fig. 3, confirms that the nanorods are composed of Zn, the C peak is from the silicone oil and the Si signal is attributed to the silicon wafer. No evidence of other impurities is found.

### Crystalline structure of a Zn nanorod

**Figure 4:**
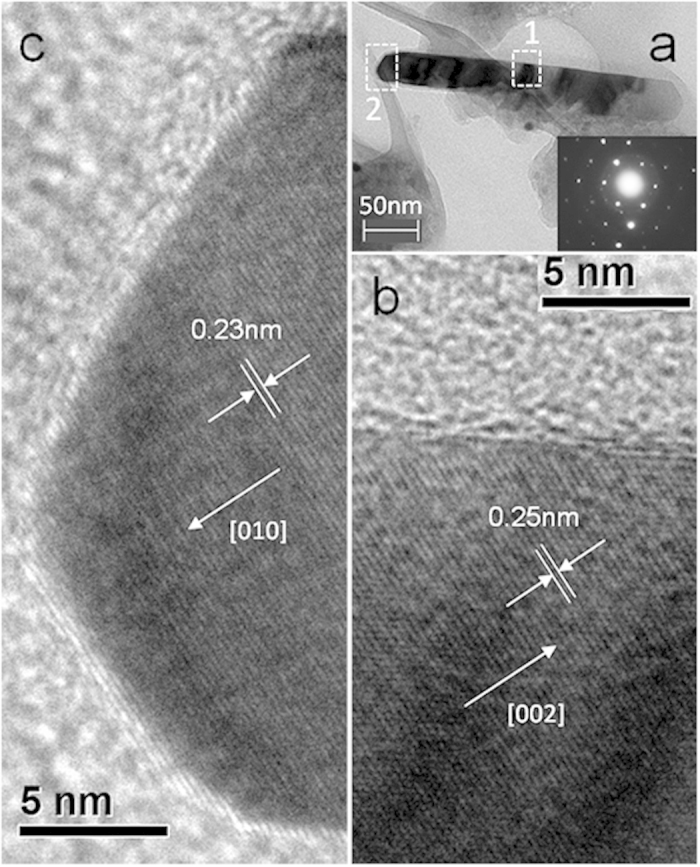
T**EM images of a Zn nanorod.** (**a**) Low-magnification TEM image of a Zn nanorod. The inset is the selected-area diffraction pattern of the nanorod in area 1. (**b**,**c**) are high-resolution TEM images of areas 1 and 2 marked in (**a)**, respectively. The white arrows show the lattice directions of the Zn nanorod.

In order to understand the microstructure of the Zn nanorods, transmission electron microscope (TEM) measurement was employed. The nanorod shown in Fig. 4a possesses two distinct tips with every corner angle of 120 degree, which is an important characteristic of single crystals. From the selected-area diffraction pattern of the nanorod in area 1 (see the inset of Fig. 4a), it is convincible that the Zn nanorod is a single crystal. In the high-resolution TEM images (Fig. 4b,c), clear lattice fringes can be observed, which further confirm that the Zn nanorods formed on the oil surfaces are crystals. From the lattice plane distances, we may deduce the atomic planes of hexagonal Zn. Within the uncertainty of our measurements, the lattice spacing of the planes in Fig. 4b shows that the Zn nanorod is made of the (002) basal planes with a preferential orientation of the [001] direction, which is in good agreement with the previous work^22^,^23^. Figure 4c shows that the emerging tip is made of the (010) planes, indicating the dependence between the growth orientation and the experimental conditions, such as the crystal edge effect, dimensional influence and the movement of defects in the crystals etc.

## Discussion

The phenomenon of the one-dimensional crystal growth on oil surfaces shown in Fig. 1 is quite unusual. In general, liquid surfaces possess an isotropic characteristic and all the deposition atoms may diffuse on the two-dimensional surface randomly due to the statistical collisions with the liquid molecules^6^,^24^. The mean square diffusion displacement <Δ*r*^2^> is given by <Δ*r*^2^> = 4*D*Δ*t*, where *D* is the diffusion coefficient and Δ*t* is the time^25^,^26^,^27^. Previous work showed that the metallic atoms and atomic clusters may diffuse on the oil surfaces with very large coefficient *D* compared with that on solid substrates^6^. Therefore compact clusters and ramified aggregates with amorphous or polycrystal microstructures are usually formed on liquid surfaces^10^,^11^. Here our investigation proves that, by adjusting proper experimental parameters, uniform Zn crystal nanorods with tunable size can also be fabricated on the isotropic liquid substrates.

In principle, the advantages of growing crystals on liquid surfaces are obvious: (1) it is not required that the lattices of the crystals match the microstructures of the liquid surfaces, in other words, various crystals may grow on liquid surfaces without the obstacle of lattice mismatch; (2) since liquid surfaces may be considered as a quasi-free sustained substrate, there is nearly no internal stress in the atomic clusters and crystals on the liquid surfaces; (3) the interaction between the deposited atoms and liquid molecules is weak and therefore the atoms are able to diffuse freely on the liquid surfaces. In this case, the mean area which the atoms can visit in time Δ*t*, i.e., <Δ*r*^2^>, is large compared with that on solid substrates. It is believed that the characteristic adhesion between Zn atoms and the liquid surface must play an important role in growing the crystals.

On the other hand, from a thermodynamic point of view, the (002) surface of Zn crystal is a high-energy plane for hexagonal crystals owing to the close packing effect. Therefore, the [002] direction would be the first preferential growth direction in vapor growth^23^. Besides, the [010] direction has also been proved to be another preferential growth direction for Zn crystal^28^. Compared with that on solid substrates, this orientated growth phenomenon may become more obvious on the isotropic and quasi-free sustained substrates, which gives the explanation for the results shown in Fig. 4b,c.

Therefore we propose that the liquid substrate effect mentioned above and the preferential growth direction of the hexagonal structure crystal together result in the growth of the Zn crystal nanorods (see Figs 1 and 2) on silicone oil surfaces.

The growth process of the Zn crystal nanorods may be depicted as follows: First, precursor Zn atoms nucleate on the oil surface and form a seed nanocrystal. In the subsequent stage, the seed nanocrystal diffuses in the area <Δ*r*^2^> during time Δ*t*. When the seed nanocrystal meets with other Zn atoms, they may irreversibly adhere to each other. After that, the Zn atoms may diffuse along the nanocrystal surface until the lowest surface energies are reached. If the seed nanocrystal forms a stable side facet, crystallization is driven along the preferential growth direction. It should be emphasized that the growth of the nanocrystal will increase the area <Δ*r*^2^>, which results in the aggregation of more Zn atoms along the preferential growth direction. Finally, an uniform Zn crystal nanorod forms on the isotropic liquid surfaces.

If the description above is correct, supposing the length increment and radius of a nanorod are Δ*L* and *r* = *W*/2, respectively, we have 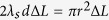, then 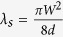, where *λ*_*s*_ is the effective surface diffusion lengths on the oil substrate^29^,^30^. For the nanorod shown in the inset of Fig. 2b (W = 27 nm, *d* = 8.0 nm), we obtain 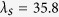 nm. As mentioned above, the most probable width range of the nanorods is 20–40 nm corresponding to 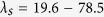 nm, which gives an explanation for the areal distribution density of the nanorods shown in Fig. 1.

Since the coefficient *D* and *λ*_*s*_ exhibit statistical characteristics, which are mainly determined by the nature of the liquid surface at temperature *T*, all the geometrical parameters of the uniform Zn crystal nanorods, including the length, width, thickness, location and orientation, should be irregularly distributed, which is in good agreement with that presented in Figs 1 and 2.

The experimental evidence above suggests a new aggregation and crystallization mechanism on liquid substrates. The key point is the interaction between the metallic atoms and the liquid molecules. Therefore, appropriate liquid materials and experimental conditions should be selected for different purposes.

In conclusion, the experimental result above provides a new and simple way to fabricate one-dimensional Zn crystals, which are important precursors for the preparation of Zn-based semiconductors^22^, such as ZnO, ZnS, ZnSe, ZnTe and so on. Furthermore, we may predict that, if the experimental conditions (such as the deposition rate, ambient temperature, surface tension of the liquid surface etc.) are appropriate, the growth of other metallic crystals (at least the crystals with the hexagonal structure, such as Be, Mg, Cd etc.) on different liquid surfaces is also possible.

## Methods

### Sample fabrication

In our experiment, commercial silicone oil (Dow Corning 705 Diffusion Pump Fluid) was chosen as the liquid substrate due to its low vapor pressure (~10^−10 ^mbar) at room temperature. The silicone oil was uniformly coated on a 10 × 10 mm^2^ ground glass with a resulting film thickness of around 0.5 mm. Zn (99.99+%, Alfa Aesar) was deposited onto the silicone oil surface at room temperature (*T* = 20±2 °C) by thermal evaporation under high vacuum (2.0 × 10^−6 ^mbar). The nominal deposition rate *f* = 0.01 nm/s and Zn film thickness *d* = 8.0 nm, which were measured by a quartz crystal microbalance located near the substrate. The sample was then removed from the evaporation chamber 30 minutes after deposition.

### Measurements

For SEM (SUPRA 55) measurement, the Zn nanorods were transferred from silicone oil surface to a polished single crystalline silicon wafer through the following procedures: (i) a polished silicon wafer pretreated by ultrasonic and absolute ethyl alcohol was pressed on the sample surface for 20 minutes, so that the Zn nanorods could adhere to the silicon wafer surface tightly; (ii) the sample covered with the silicon wafer was then soaked in acetone for 10 minutes; (iii) the nanorods-covered silicon wafer was separated from the substrate carefully and then kept it in fresh acetone for another 5 minutes; (iv) the silicon wafer was then transferred into absolute ethyl alcohol for 10 minutes; (v) the silicon wafer was picked out of the absolute ethyl alcohol and exposed to the infrared oven lamp for 5 minutes. It should be mentioned that, after the manipulation of the five procedures above, the relative locations of all the Zn nanorods would not change obviously. The compositions of the nanorods were investigated by EDS. On the other hand, a TEM (JEM-2010) was also used to measure the microstructure of the Zn nanorods, which were previously shifted to a copper grid with ultrathin carbon film.

## Additional Information

**How to cite this article**: Lu, C. *et al*. One-dimensional Growth of Zinc Crystals on a Liquid Surface. *Sci. Rep.* **6**, 19870; doi: 10.1038/srep19870 (2016).
